# Circular RNA circNID2 Promotes Bovine Adipogenesis via miR-339a/NNAT

**DOI:** 10.3390/biom16081113

**Published:** 2026-07-30

**Authors:** Yue Liu, Mengyang Zhang, Zhuoyuan He, Li Sun, Yangyang Bai, Chuanying Pan, Xiaomei Sun, Yang Li, Enhui Jiang, Xianyong Lan

**Affiliations:** 1 Key Laboratory of Animal Genetics, Breeding and Reproduction of Shaanxi Province, College of Animal Science and Technology, Northwest A&F University, Yangling 712100, China; liuyue527@nwsuaf.edu.cn (Y.L.); zhangmy2023@nwafu.edu.cn (M.Z.); hzy001@nwafu.edu.cn (Z.H.); 2024050452@nwafu.edu.cn (L.S.); baiyangyang2022@nwafu.edu.cn (Y.B.); chuanyingpan@nwafu.edu.cn (C.P.); yangli2001@nwsuaf.edu.cn (Y.L.); 2 Key Laboratory for Animal Genetics, Breeding, Reproduction and Molecular Design of Jiangsu Province, College of Animal Science and Technology, Yangzhou University, Yangzhou 225009, China; sunxm@yzu.edu.cn

**Keywords:** circNID2, adipose, coding, ceRNA

## Abstract

Adipose tissue development significantly influences meat quality and economic traits in beef cattle. In this study, we identified a novel circular RNA derived from two exons (exons 3 and exons 9) of the *NID2* gene, designated as circNID2, which exhibits high structural stability and marked upregulation during *bovine* adipocyte differentiation. Functional assays demonstrated that circNID2 suppresses the proliferation and apoptosis of *bovine* preadipocytes while robustly promoting adipogenic differentiation and lipid accumulation. Although containing several putative open reading frames, circNID2 lacks protein-coding potential. Mechanistically, circNID2 functions as a competing endogenous RNA by sponging miR-339a, thereby relieving the post-transcriptional repression of its downstream target, Neuronatin (*NNAT*). Functional rescue experiments further validated that circNID2 partially neutralizes the regulatory effects of miR-339a on preadipocyte proliferation, apoptosis, and differentiation. Collectively, these findings demonstrate that circNID2 regulates bovine preadipocyte function through the newly established circNID2/miR-339a/NNAT axis. This study provides novel insights into the post-transcriptional mechanisms governing mammalian adipogenesis and highlights circNID2 as a potential molecular target for improving fat deposition traits in cattle.

## 1. Introduction

Adipose tissue acts as more than just a major organ for energy storage. It also serves as a critical regulator of endocrine, immune, and metabolic homeostasis. In production practice, how much fat an animal deposits largely determines the quality of its meat. Specifically, the amount of intramuscular fat (IMF) controls key characteristics like marbling, tenderness, juiciness, and flavor. These qualities in turn affect how valuable the beef is and whether consumers will accept it [1,2]. The formation of adipose tissue is a highly coordinated biological process involving multiple sequential events, including preadipocyte proliferation, mitotic clonal expansion, differentiation, and adipocyte maturation, while apoptosis primarily contributes to the regulation of adipose tissue homeostasis and remodeling [3]. In this regulatory landscape, non-coding RNAs (ncRNAs), particularly circular RNAs (circRNAs), have emerged as important post-transcriptional regulators that modulate these cellular transitions [4]. Given their economic value and the direct link between fat development and production traits, cattle serve as an ideal model for studying adipogenesis. Furthermore, bovine adipocyte differentiation shares conserved regulatory mechanisms with other mammals, rendering cattle a valuable model for exploring the molecular networks of fat deposition. Hence, a systematic dissection of the molecular mechanisms controlling adipocyte fate is critically needed, which will not only advance fundamental adipocyte biology but also provide vital biological insights for breeding high-quality beef cattle and enhancing livestock production efficiency.

With a longer intracellular half-life than linear RNAs, circRNAs exhibit enhanced molecular stability, which contributes to their potential as promising biomarker candidates and functional regulators in mechanistic studies [2,5]. Moreover, circRNAs exhibit high evolutionary conservation and tissue-specific expression patterns. circRNAs actively participate in various aspects of cellular life, ranging from cell proliferation to metabolic homeostasis [6]. The known primary mechanisms of circRNA include acting as competing endogenous RNA (ceRNA) to bind microRNA (miRNA), interacting with RNA-binding proteins, regulating host gene expression, and translating functional peptides under specific conditions [7,8,9]. While increasing evidence indicates that some circRNAs may regulate livestock myogenesis and adipogenesis through a ceRNA mechanism by interacting with miRNAs and modulating downstream gene expression, the functional significance of this regulatory mode remains dependent on the specific circRNA-miRNA-mRNA context [10]. Beyond this well-trodden non-coding path lies a vast, yet largely unchartered, molecular territory governing the non-canonical translation mechanisms of these circular transcripts.

miRNAs represent another important group of ncRNAs that actively control cell fate. Mechanistically, these concise transcripts achieve target-gene silencing by scanning and anchoring to complementary sequences within the 3′ untranslated regions of messenger RNAs. This sequence-specific engagement destabilizes the architectural integrity of the transcript or physically impedes the ribosomal machinery, effectively bottlenecking protein synthesis [11]. Among the many miRNAs involved in adipocyte development, miR-143 and miR-378 serve as typical examples [12,13]. By comparison, miR-339 has been found to regulate cell growth only in tumor contexts, and its potential roles in adipocytes have not been systematically investigated. Standing at the intersection of epigenetic programming and metabolic control, Neuronatin (*NNAT*) represents a critically imprinted gene that directly dictates the functional plasticity of adipocytes. Rather than acting as a passive downstream player, this architecturally distinct locus serves as a metabolic hub, fine-tuning the enzymatic cascades that orchestrate cellular lipid accrual [14]. However, whether circNID2 participates in bovine adipocyte differentiation through a miR-339a-mediated ceRNA regulatory mechanism involving *NNAT* remains unknown.

In our previous study, we performed circRNA sequencing on bovine preadipocytes at different differentiation stages. Tailored to the dynamic transcriptional landscape of bovine adipogenesis, our preliminary screen unveiled an uncharacterized back-splicing event spanning exons 3 and 9 of the NID2 gene. This non-canonical processing yields a differentially expressed circular transcript, which we have designated herein as circNID2. Preliminary results showed that circNID2 is significantly up-regulated during adipocyte differentiation, suggesting a potential regulatory role. While the linear transcript of nidogen 2 (*NID2*) is known to be involved in tissue remodeling, the function of its circular form in bovine adipocytes has not been reported. Therefore, this study aimed to characterize the biological function of circNID2 during bovine adipocyte differentiation and investigate the potential molecular mechanisms underlying its regulation, with a particular focus on the possible involvement of miRNA-mediated regulatory networks. Consequently, our study not only examined whether circNID2 can encode a protein but also revealed how it functions as a ceRNA at the molecular level. These findings will help reveal the molecular mechanisms of bovine fat development.

## 2. Materials and Methods

### 2.1. Ethics Statement

Tissue samples including heart, liver, lung, kidney, intestine, skeletal muscle, and adipose were collected from three individual cattle. Immediately after collection, each sample was placed into liquid nitrogen before being transferred to a −80 °C freezer. The study followed the guidelines of the “Management of Laboratory Animal Affairs” established by the Ministry of Science and Technology, with ethical approval from the Ethics Committee of Northwest A&F University (DK2022032).

### 2.2. Primary Adipocyte Isolation and Culture

The fetal bovine was cleaned with 75% ethanol, and then subcutaneous fat was harvested under sterile conditions for later use. Tissue pieces were rinsed twice with 75% ethanol and twice with PBS containing 2% penicillin-streptomycin, then minced in a dish with a small volume of complete medium (DMEM/F-12 with 20% FBS and 1% antibiotics). The minced tissue was digested in 0.2% collagenase I at 37 °C for 2 h with agitation. Following centrifugation, the pellet was washed with pre-warmed PBS and cultured in complete medium at 37 °C in 5% CO_2_. Cells were passaged upon reaching about 80% confluence.

### 2.3. CircRNA Validation

To confirm the circular nature of the candidate circRNAs, three assays were performed. Back-splicing junction identification. PCR was performed with convergent and divergent primers on gDNA and cDNA. Convergent primers yielded gDNA and cDNA products. Divergent primers generated a specific band only from cDNA. This band was gel-purified and Sanger-sequenced, confirming the exact circular junction site, thus verifying circNID2 formation.

RNase R digestion assay. To verify exonuclease resistance, 2 μg of total RNA was incubated with 2 U/μg of RNase R at 37 °C for 15 min, with an equal aliquot treated without RNase R as a control. After digestion, the abundance of circNID2 and its linear mRNA NID2 counterpart was measured by RT-qPCR.

Actinomycin D stability assay. To evaluate transcript stability, cells were treated with actinomycin D (2 μg/mL) and harvested at 0, 2, 4, 8, 10, 14 and 24 h after treatment. Total RNA was extracted at each time point, and RT-qPCR was performed to monitor the decay of circNID2 and linear mRNA NID2.

### 2.4. Vector Construction and Transfection

To overexpress circNID2 in cells, we inserted the full-length circNID2 cDNA into the PCD25 vector to generate the circNID2 overexpression construct. miR-339a mimics were synthesized by GenePharma (Shanghai, China). Using Lipofectamine 2000 (Invitrogen, Waltham, MA, USA), all vectors were transfected into adipocytes following the manufacturer’s protocol, and the transfected cells were then used for subsequent experiments. Cells were transfected with circNID2 overexpression vector, miR-339a mimic with corresponding empty vector used as controls. Cells were harvested 48 h after transfection for subsequent analyses.

### 2.5. Real-Time Quantitative Polymerase Chain Reaction (RT-qPCR)

Total RNA was extracted using TRIzol method. Then, 1 μg of RNA was converted into cDNA using PrimeScript RT Enzyme (Yeasen, Shanghai, China). SYBR qPCR Master Mix (Vazyme, Nanjing, China) was employed for RNA amplification and quantification. The reaction mixture consisted of 13 µL, including 0.5 µL of upstream and downstream primers each, 6.5 µL of reaction mix, 0.5 µL of ddH_2_O, and 5 µL of cDNA diluted 40-fold. The qPCR conditions consisted of an initial denaturation step at 95 °C for 30 s, then 40 cycles of 95 °C for 10 s and 60 °C for 30 s for annealing and extension. All primers were validated by melt curve analysis, and their sequences are listed in Table 1. GAPDH served as the reference gene to normalize CT values. The ΔΔCT method was applied to determine target gene expression alterations, which were expressed as fold changes (FC = 2^−ΔΔCT^).

### 2.6. Cell Proliferation

Following transfection, primary bovine adipocytes were plated in 96-well culture plates and maintained in growth medium for 24 h. Ten independent replicates were used per treatment group. After this period, each well received 10 μL of CCK-8 reagent (Solarbio, Beijing, China) and was incubated for 2 h at 37 °C. An enzyme marker (BioTek, USA) measured the absorbance at 450 nm. Preadiocyte mitotic activity was monitored via the tracking of active DNA synthesis using a 5-ethynyl-2′-deoxyuridine (EdU) labeling toolkit (RiboBio, Guangzhou, China). Briefly, replicating cells were exposed to the EdU substrate, allowing the nucleotide analog to incorporate into the newly synthesized genomic DNA strands. The subsequent azide-alkyne cycloaddition was executed to conjugate the fluorophore, and the resulting fluorescently labeled nuclei were captured and visualized utilizing an inverted fluorescence microscope.

### 2.7. Apoptosis Analysis

To quantify programmatic cell death, genomic DNA fragmentation was tracked via a Terminal deoxynucleotidyl transferase (TdT)-mediated dUTP nick-end labeling (TUNEL) analytical suite (RiboBio, Guangzhou, China). Mechanistically, the exogenously supplied TdT enzyme catalyzed the addition of fluorescein-dUTP onto the exposed 3′-hydroxyl termini of cleaved DNA strands within apoptotic nuclei. The resulting localized fluorescent clusters were captured using an inverted fluorescence microscope. To ensure statistical stringency, the entire procedural workflow was executed across three independent biological cohorts.

### 2.8. Oil Red O Staining

Intracellular lipid loading was visualized at the 9-day post-differentiation milestone using a lipophilic Oil Red O (Sigma, Ronkonkoma, NY, USA). The specific incorporation of the dye into the hydrophobic core of cellular vacuoles generated a robust histochemical readout. The stained cultures were then processed immediately for subsequent microscopic imaging and extraction. For quantification of fat droplet staining, the lipid droplets were dissolved in 100% isopropanol, and absorbance was read at 510 nm [15].

### 2.9. Dual-Luciferase Reporter Assay

To validate the physical interaction between circNID2 and miR-339a, the constructs were generated by inserting either the wild-type sequence of circNID2 containing the predicted miR-339a binding site (circNID2-WT) or a mutant sequence (circNID2-MUT) into the multiple cloning site of the psiCHECK-2 backbone. The circNID2-MUT construct was generated by replacing the putative miR-339a binding sequence GACAGGGA with the mutant sequence CTGTCCCT, thereby disrupting the predicted binding site. Following sequence validation, these recombinant vectors were introduced into an engineered HEK293T cellular background characterized by sustained miR-339a hyper-expression. At the 24 h post-transfection milestone, cellular architectures were disrupted using a specialized lysis formulation to liberate the intracellular reporter enzymes. The sequential biocatalytic luminescence emitted by Renilla and Firefly luciferases was subsequently captured and resolved using a microplate detection system (BioTek, Winooski, VT, USA).

### 2.10. Statistical Analyses

All cellular and molecular measurements derived from this investigation were mathematically compiled as mean values paired with the standard deviation (SD) from at least three separate biological replications. Mathematical modeling of the experimental boundaries was orchestrated via the SPSS 22.0 computational platform (SPSS, Chicago, IL, USA). For binary comparisons involving isolated treatments, the parametric Student’s *t*-test was implemented. To resolve variance across complex multi-group matrices without inflating type I error rates, a global one-way ANOVA followed by Tukey’s post hoc comparison was systematically deployed. The boundary dividing mathematical stochasticity from biological significance was restricted to a probability value of less than *p* < 0.05. * indicates *p* < 0.05, ** indicates *p* < 0.01.

## 3. Results

### 3.1. Characterization and Structural Validation of Novel circNID2 in Bovine Preadipocytes

**Figure 1:**
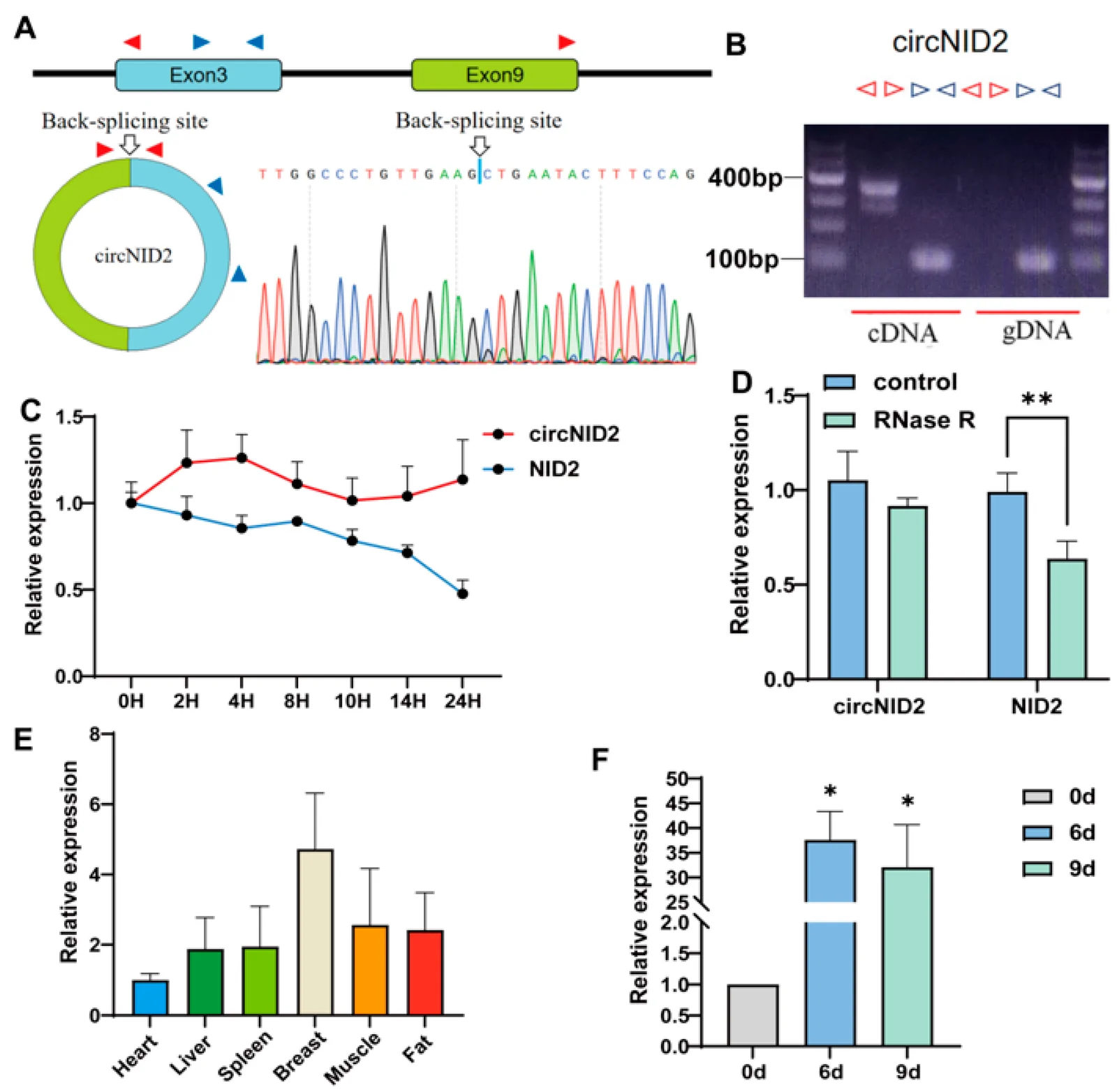
Characterizations of circNID2. (**A**) Schematic of the structure of circNID2. The arrow indicates the Back-splicing site. (**B**) Forward and reverse primers validate the circular structure of circNID2, where red icons indicate reverse primers and blue icons indicate forward primers. (**C**) RT-qPCR for the circNID2 and NID2 treated with actinomycin D. (**D**) Detection of circNID2 and NID2 mRNA expression in bovine precursor adipocytes after RNase R treatment. (**E**) Analysis of circNID2 tissue expression profiles. (**F**) Expression of circNID2 in bovine precursor adipocytes at different periods of differentiation. * indicates *p* < 0.05, ** indicates *p* < 0.01.

To screen for candidate circRNAs associated with adipogenic differentiation, our prior transcriptome-wide sequencing across distinct preadipocyte differentiation was performed. Based on the differential expression patterns (|log2FC| > 1.5, *p* < 0.05) and subsequent validation, a circRNA derived from the Nidogen 2 (NID2) locus, designated as circNID2, was selected as a candidate for further investigation. Head-to-tail junctional splicing was validated at the nucleotide level through outward-facing divergent primer amplification coupled with definitive Sanger sequencing, mapping the circular junction precisely to the ligation of NID2 exon 3 and exon 9 (Figure 1A). Direct genomic evaluation via agarose gel electrophoresis revealed that while convergent primers amplified both genomic DNA (gDNA) and complementary DNA (cDNA) templates, the divergent primer pair yielded a singular, specific amplicon strictly within the cDNA pool, confirming that the back-spliced interface stems from post-transcriptional looping rather than genomic duplication (Figure 1B).

Given that true circular transcripts bypass standard exonucleolytic decay, we next challenged the architectural resilience of circNID2. Transcription blockade via Actinomycin D exposure demonstrated that circNID2 maintained a significantly prolonged half-life relative to the rapidly decaying linear NID2 messenger RNA, indicating high baseline metabolic stability (Figure 1C). This topology was firmly cross-validated by incubating total RNA with RNase R exonuclease; the linear NID2 transcript underwent wholesale clearance, whereas circNID2 exhibited profound digestion resistance (Figure 1D). Spatial profiling across an expanded bovine tissue panel illustrated a broad, ubiquitous expression landscape, suggesting a systemic physiological relevance (Figure 1E). Crucially, monitoring circNID2 accumulation kinetics during adipogenic chronological progression revealed a synchronized pattern (Figure 1F). Taken together, these rigorous structural and spatial metrics establish circNID2 as a highly stable, non-canonical spliced entity with a potential regulatory mandate in bovine adipocyte commitment.

### 3.2. circNID2 Promotes Adipogenic Differentiation While Inhibiting Proliferation and Apoptosis in Bovine Preadipocytes

**Figure 2:**
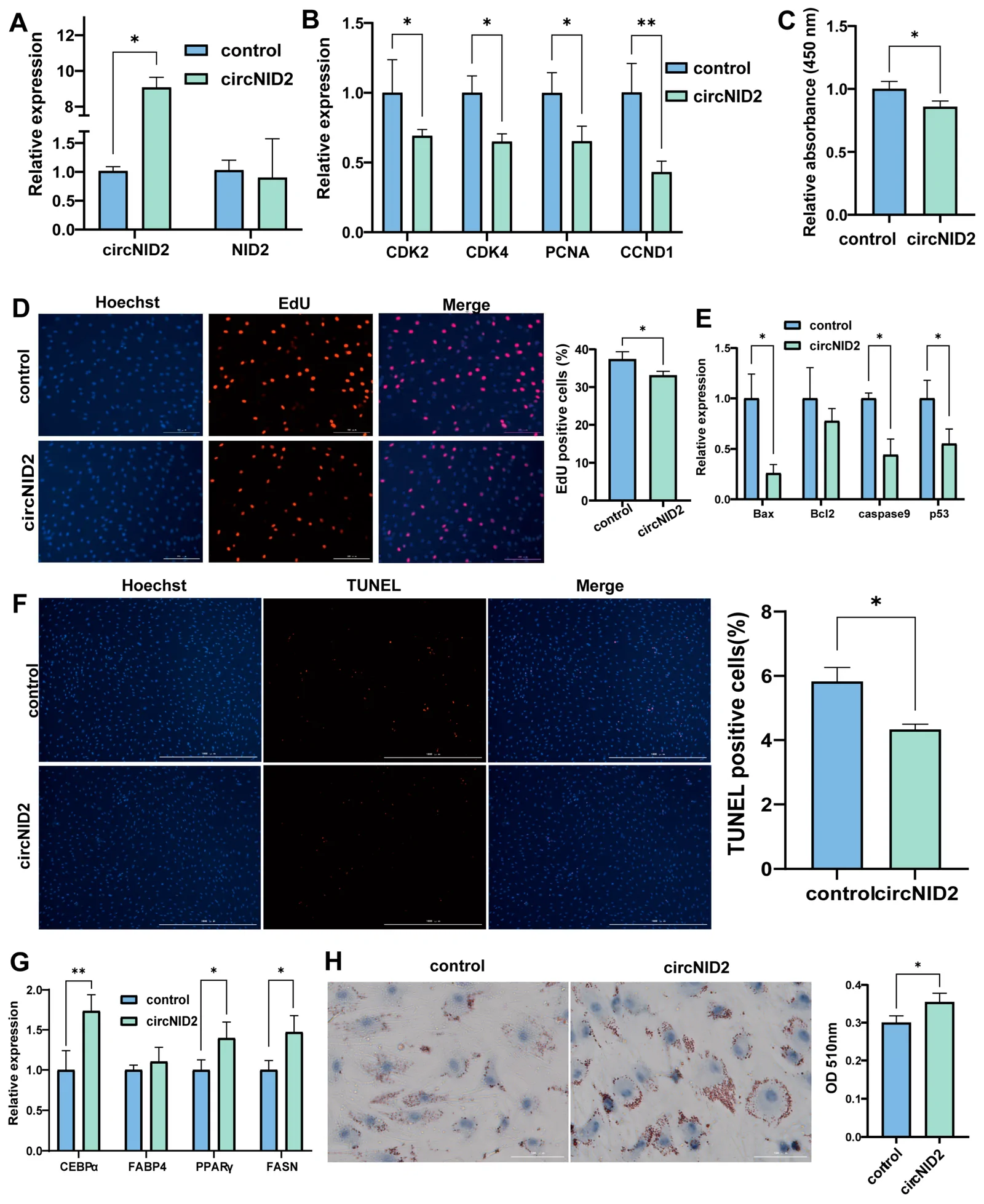
circNID2 modulates proliferation, apoptosis, and differentiation in bovine primary preadipocytes. (**A**) RT-qPCR was performed to measure the relative expression of the host gene NID2 in preadipocytes following transfection with the circNID2 overexpression vector (circNID2). (**B**) RT-qPCR analysis of proliferation marker genes following circNID2 overexpression. (**C**) CCK-8 assay measuring cell viability after circNID2 overexpression or interference. (**D**) EdU incorporation assay and quantification. Scale = 200 μm. (**E**) RT-qPCR analysis of apoptosis-related genes after circNID2 overexpression. (**F**) TUNEL incorporation assay and quantification. Scale = 1000 μm. (**G**) RT-qPCR analysis of differentiation marker genes after circNID2 overexpression. (**H**) Staining of differentiated preadipocytes with Oil Red O and subsequent measurement of lipid droplet content. Scale = 100 μm. * indicates *p* < 0.05, ** indicates *p* < 0.01.

Armed with a validated circNID2 overexpression platform engineered within bovine primary preadipocytes (Figure 2A), we sought to map its definitive phenotypic footprint across a spectrum of vital cellular activities. In the context of population dynamics, the expression profiles of key proliferation-responsive targets were initially mapped, showing a significant, widespread reduction in their transcriptional velocity upon circNID2 hyper-activation (Figure 2B). This negative regulatory pattern was cross-validated through real-time metabolic monitoring via a CCK-8 assay system, which uncovered a significant, time-dependent restriction on overall cell viability (Figure 2C). To gain high-resolution spatial evidence of this growth arrest, DNA synthesis was directly monitored through fluorometric EdU tracking. The quantitative readout revealed that circNID2 overabundance effectively locked the cells out of active synthesis, significantly minimizing the percentage of EdU-positive dividing centers (Figure 2D). Interestingly, this suppressed growth kinetics did not steer the cells toward homeostatic breakdown. On the contrary, genomic monitoring showed that circNID2 effectively shielded preadipocytes by significantly downregulating essential apoptosis-inducing factors (Figure 2E). This protective behavior was visually fixed through a fluorometric TUNEL cleavage assay, where the occurrence of localized chromosomal fragmentation was markedly lowered (Figure 2F). These dual lines of evidence confirm that circNID2 establishes an anti-proliferative, pro-survival state in bovine preadipocytes.

Beyond supervising foundational homeostatic cellular behaviors, we further reasoned that circNID2 might dictate the specialized phenotypic transition from undifferentiated progenitors to mature, lipid-laden adipocytes. Real-time quantitative assessment illustrated that the accumulation of key adipogenic marker genes rose sharply and significantly in the presence of excess circNID2 (Figure 2G). This robust upregulation at the transcript level was seamlessly mirrored at the morphological level by downstream histochemical processing. Oil Red O staining of the differentiated cultures provided intuitive visual evidence, showing that circNID2 hyper-activation induced a substantial, dense accumulation of neutral triglycerides inside the mature cell vacuoles (Figure 2H). Summarizing these findings, our data indicate that circNID2 functions as a critical switch in bovine fat development, simultaneously repressing the metabolic drain of proliferation and apoptosis to prioritize and accelerate the macromolecular machinery driving adipogenic commitment.

### 3.3. circNID2 Lacks Protein-Coding Capacity

**Figure 3:**
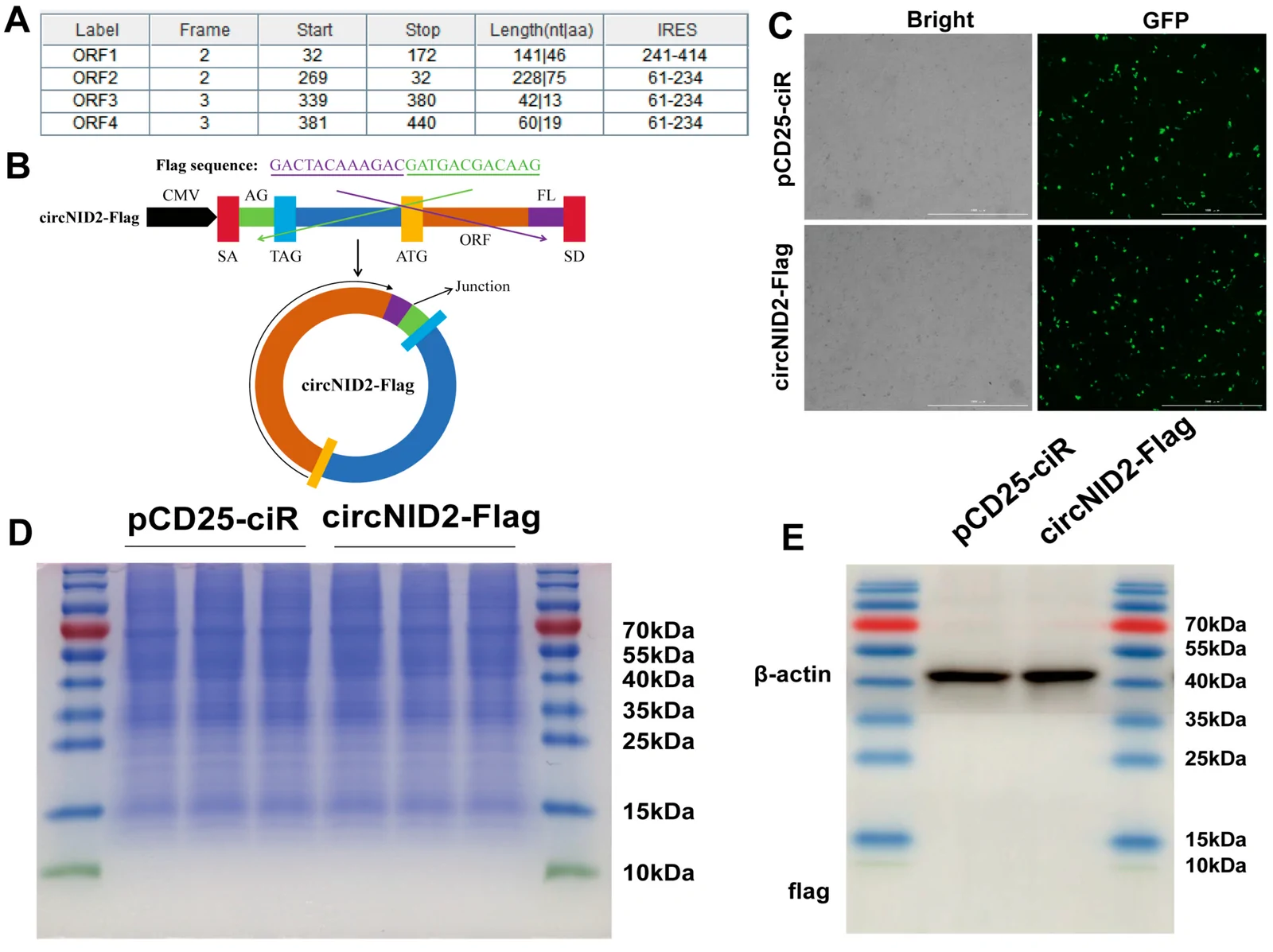
Prediction and experimental validation of the coding potential of circNID2. (**A**) Information on the specific details of four ORFs predicted by circPrimer 2.0 software. (**B**) Design of a vector with a Flag tag. (**C**) pCD25-ciR vector and circNID2-Flag vector transfection fluorescence. Scale = 1000 μm. (**D**) Coomassie brilliant blue staining is used to detect the total protein expression in HEK293T cells. (**E**) Use a Flag-tagged antibody to detect the NID2-75aa-Flag protein. β-actin serves as the internal reference, with a protein size of 42 kDa. The original Western blot images are available in the Supplementary Materials.

Given that some circRNAs possess hidden translational capabilities, we next investigated whether circNID2 could likewise encode a functional peptide. Sequence analysis identified multiple potential open reading frames (ORF), among which the longest ORF was predicted to encode a peptide of approximately 75 amino acids (Figure 3A,B). To evaluate its translational potential, a circNID2-Flag reporter vector was constructed in which the Flag tag could be expressed only after successful circularization of circNID2 (Figure 3C). Following transfection into 293T cells, fluorescence microscopy confirmed successful transfection (Figure 3D). However, neither Western blotting nor Coomassie Brilliant Blue staining detected the predicted protein product (Figure 3E), indicating that circNID2 lacks protein-coding capacity.

### 3.4. circNID2 Functions as a Molecular Sponge for miR-339a

**Figure 4:**
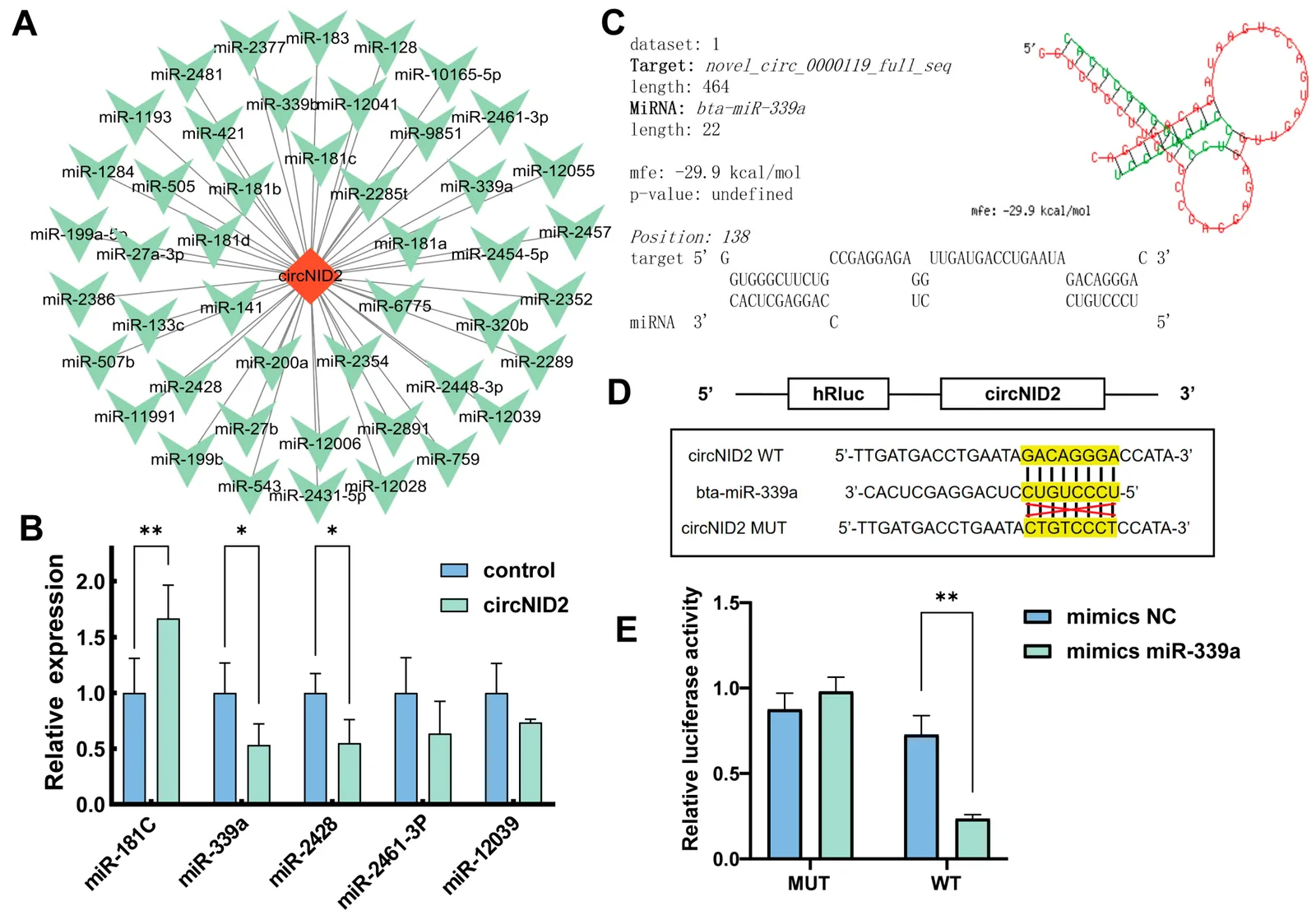
circNID2 directly interacts with miR-339a as a competing endogenous RNA (ceRNA). (**A**) Utilize Cytoscape v3.10.0 software to draw a network diagram of miRNAs binding to circNID2 based on the prediction results. (**B**) RT-qPCR was used to examine how circNID2 overexpression affects the expression of various miRNAs. (**C**) Prediction of the secondary structure of miR-339a. (**D**) Illustration showing the binding sites between miR-339a and the dual-luciferase reporter constructs circNID2-WT and circNID2-MUT. (**E**) Relative luciferase activities of circNID2-WT and circNID2-MUT after co-transfection with miR-339a mimics or negative control (NC) mimics. * indicates *p* < 0.05, ** indicates *p* < 0.01.

To uncover the precise molecular mechanism driving the downstream phenotypes of circNID2, we investigated its capacity to operate as a competitive regulatory decoy for microRNAs. In silico screening utilizing the RNAhybrid alignment utility provided an initial blueprint of potential target networks by identifying vulnerable non-coding complements (Figure 4A). Subsequent intracellular quantification demonstrated that shifting the homeostatic equilibrium via ectopic circNID2 delivery severely disrupted the availability of its predicted targets, prompting a significant reduction in the expression metrics of both miR-339a and miR-2428 (Figure 4B). Strategic base-pairing analysis further refined this field, illustrating that the structural docking between circNID2 and miR-339a possessed superior conformational stability and free-energy characteristics (Figure 4C).

We cross-examined this binding axis through a definitive gain-of-function luciferase tracking configuration to rule out indirect transcriptional artifacts (Figure 4D). The physical engagement of the two non-coding species was confirmed when the introduction of exogenous miR-339a mimics specifically paralyzed the translation of the circNID2-WT reporter construct, driving a sharp decline in measurable luminescence. This regulatory block was structurally dependent on sequence fidelity. The engineered mutant variant circNID2-MUT, which lacked the necessary target seed configuration, proved entirely resistant to miR-339a-mediated suppression and maintained baseline bioluminescent emission (Figure 4E). These data establish a clear biochemical mechanism, demonstrating that circNID2 serves as a functional molecular sink that effectively sequesters miR-339a to guide bovine cell fate.

### 3.5. circNID2 Regulates Bovine Adipocyte Functions via Sponging miR-339a

**Figure 5:**
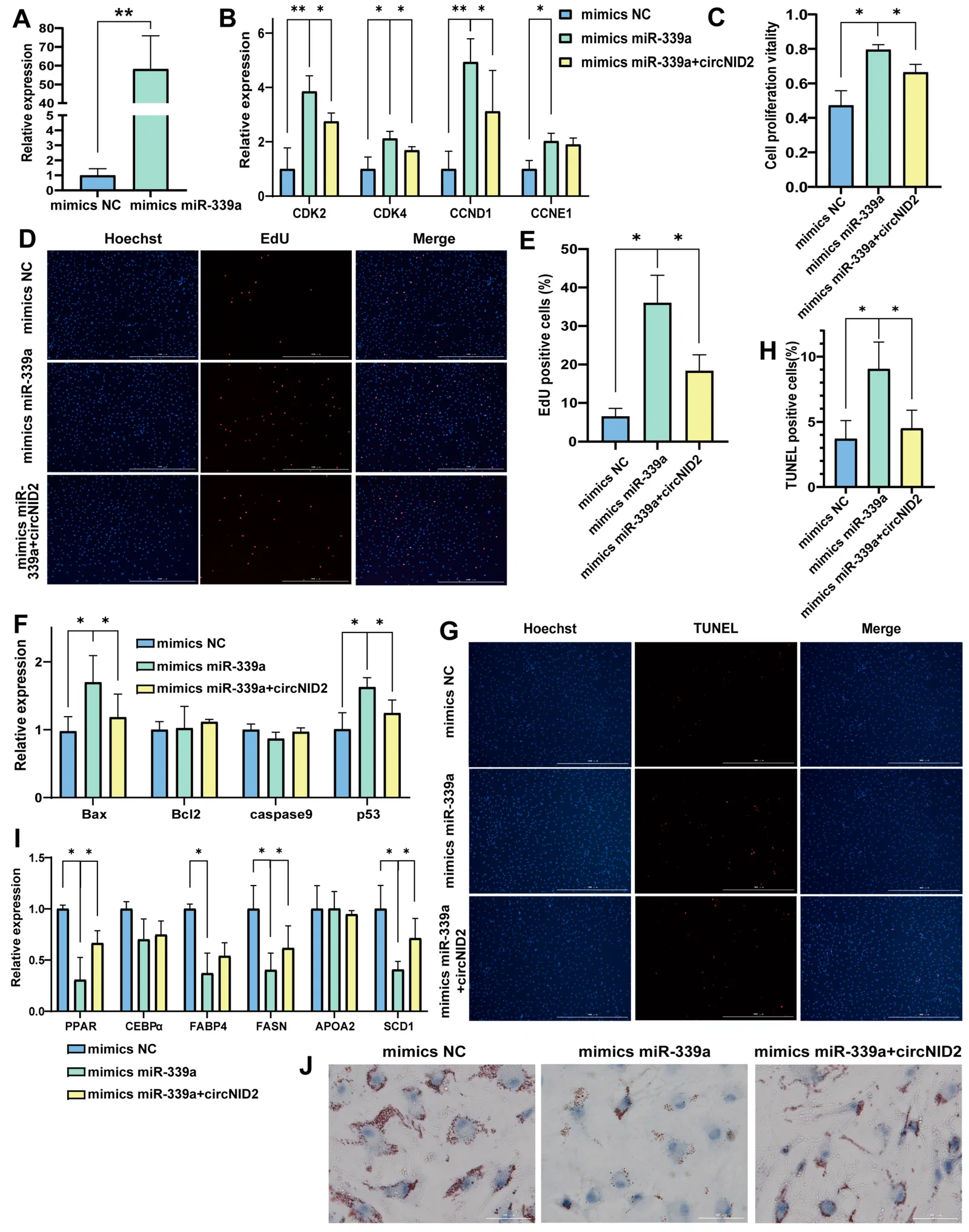
circNID2 rescues miR-339a-induced suppression of proliferation and adipogenesis and promotion of apoptosis. (**A**) RT-qPCR assay was conducted to verify the overexpression efficiency of mimics miR-339a. (**B**) Proliferation marker gene detection. (**C**) Colorimetric CCK-8 growth curves, (**D**,**E**) and fluorometric tracking alongside quantification of active DNA synthesis via EdU incorporation. Scale = 1000 μm. (**F**–**H**) Programmed cell death dynamics analyzed across the identical multi-factorial cohorts, showing mRNA shifts in core cell-death components (**F**), TUNEL imaging and quantitative cell counting were performed following the same three transfection conditions: NC mimics, miR-339a mimics, and co-transfection of miR-339a mimics with circNID2 (**G**,**H**). Scale = 1000 μm. (**I**) The expression of adipocyte-specific marker genes was examined by RT-qPCR in cells that received NC mimics, miR-339a mimics, or the combination of miR-339a mimics and circNID2, (**J**) and cytochemical visualization of neutral triglyceride deposition via Oil Red O staining. Scale = 100 μm. * indicates *p* < 0.05, ** indicates *p* < 0.01.

To investigate the functional relationship between circNID2 and miR-339a, miR-339a overexpression and co-transfection models were established in bovine preadipocytes. Transfection of miR-339a mimics into bovine preadipocytes confirmed high transfection efficiency, with a marked overexpression effect, allowing further experiments (Figure 5A). To determine whether circNID2 executes its biological mandate via the newly identified non-coding interface, a tripartite genetic rescue design (mimics NC, mimics miR-339a, and mimics miR-339a + circNID2) was implemented. RT-qPCR revealed that isolated miR-339a hyper-activation robustly up-regulated key cell cycle drivers, including CDK2, CDK4, CCND1, and CCNE1. Strikingly, concurrent circNID2 delivery effectively blunted this molecular acceleration (Figure 5B). This transcriptional containment seamlessly mirrored cell population behaviors. Kinetic monitoring via CCK-8 tracking demonstrated that while miR-339a overabundance forced a sharp upward shift in cumulative preadipocyte expansion, co-transfection with circNID2 severely bottlenecked this growth-promoting effect (Figure 5C). These concurrent metrics confirm that circNID2 rescues the preadipocyte proliferation profile by actively trapping and neutralizing miR-339a. This functional rescue was further visualized through EdU imaging, which intuitively illustrated that the increased proliferative activity induced by miR-339a was markedly weakened upon circNID2 co-transfection. Statistical quantification of EdU-positive cells validated these significant inter-group differences (Figure 5D,E). Combined, these data confirm that circNID2 restricts bovine adipocyte proliferation by sponging miR-339a.

To further paint a comprehensive picture of cellular fate regulated by this axis, we subsequently evaluated the joint impact of miR-339a and circNID2 on programmed cell death, focusing on core apoptosis marker genes (Bax, Bcl2, caspase9, and p53). RT-qPCR analysis revealed that Bax and p53 levels rose sharply following miR-339a overexpression, but this pro-apoptotic trend was efficiently counteracted by circNID2 co-transfection (Figure 5F). At the cellular level, fluorometric TUNEL profiling revealed that the sharp expansion of apoptotic nuclei triggered by isolated miR-339a overabundance was completely deactivated upon concurrent circNID2 introduction, successfully rescuing preadipocyte survival inside the co-transfection group (Figure 5G,H). Consequently, these results demonstrate that circNID2 protects against bovine adipocyte apoptosis through its competitive binding to miR-339a. Finally, we explored the downstream consequences on adipogenic differentiation using a combination of RT-qPCR and Oil Red O staining. Gene expression profiling indicated that miR-339a overexpression significantly repressed major adipogenesis markers, including PPARγ, CEBPα, FABP4, FASN, and SCD1 (*p* < 0.05). However, the simultaneous delivery of circNID2 effectively counteracted this transcriptional reduction (Figure 5I). Morphologically, Oil Red O staining confirmed that the severe restriction on lipid droplet biogenesis imposed by miR-339a was partially salvaged through circNID2 co-introduction (Figure 5J). Collectively, these metrics demonstrate that miR-339a acts as a negative regulator of adipogenesis, a bottleneck successfully neutralized by circNID2 acting as a molecular sponge. By doing so, it effectively alleviates the miR-339a-mediated regulation of proliferation, apoptosis, and differentiation, thereby acting as a pivotal driver of bovine adipocyte development.

### 3.6. NNAT as a Direct Target of miR-339a

**Figure 6:**
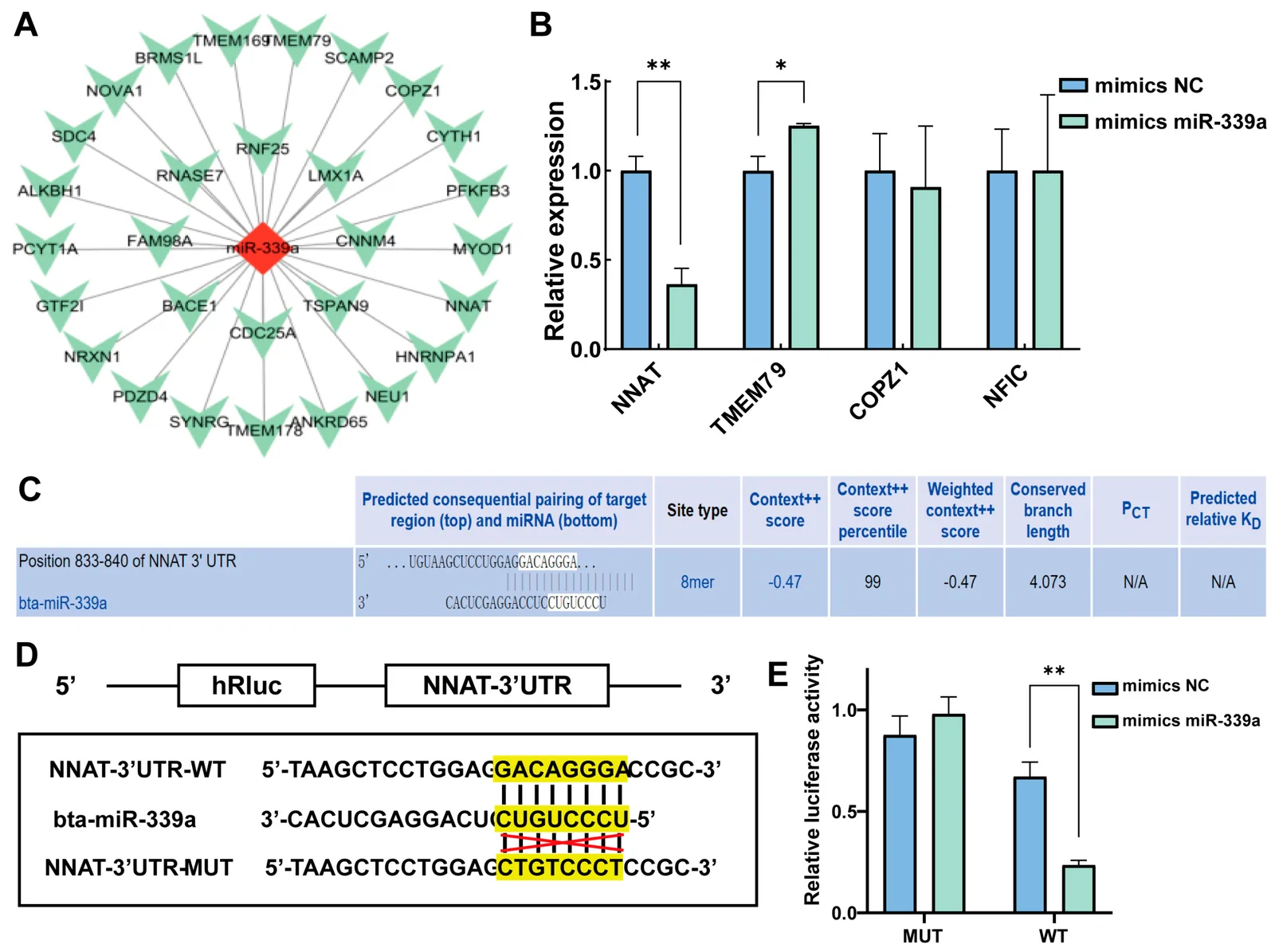
miR-339a directly targets *NNAT*. (**A**) A network diagram of predicted target genes interacting with miR-339a was constructed using Cytoscape v3.10.0 software. (**B**) The expression levels of target genes after miR-339a overexpression were detected by RT-qPCR. (**C**) The TargetScan website predicts the binding sites of miR-339a with the *NNAT* gene. (**D**) A diagram illustrating how miR-339a interacts with the wild-type (NNAT-3′UTR-WT) and mutant (NNAT-3′UTR-MUT) dual-luciferase reporter constructs. (**E**) Measurement of relative luciferase activity from NNAT-3′UTR-WT and NNAT-3′UTR-MUT reporters following separate transfection with control mimics (NC) and miR-339a mimics. * indicates *p* < 0.05, ** indicates *p* < 0.01.

To clarify the downstream molecular architecture through which the circNID2/miR-339a axis governs preadipocyte commitment, potential downstream messenger RNA configurations were cross-referenced utilizing TargetScan and miRWalk computational engines, from which an interactive regulatory topography was mapped (Figure 6A). High-scoring candidate transcripts were filtered and systematically subjected to expression validation via RT-qPCR. This initial screening isolated NNAT as a highly sensitive downstream component, displaying a profound transcriptional drop upon singular miR-339a introduction (Figure 6B).

Detailed TargetScan analysis revealed perfect complementarity between nucleotides 1–8 at the 5′ end of miR-339a and the 3′UTR of NNAT (Figure 6C). To formally validate this sequence-specific interaction, a wild-type dual-luciferase reporter plasmid, designated as NNAT-3′UTR-WT, was constructed by cloning approximately 200 bp of the NNAT 3′UTR flanking the predicted binding site into the psiCHECK-2 vector. Concurrently, a mutant construct named NNAT-3′UTR-MUT was generated by introducing complementary mutations directly into the predicted binding seed sequence (Figure 6D). Transient co-transfection profiles within HEK293T demonstrated that miR-339a hyper-activation caused a robust, statistically superior restriction of relative luciferase photon emission driven by the wild-type construct. Conversely, this catalytic silencing was entirely abolished upon seed-region mutagenesis, leaving the luminescent readout undisturbed (Figure 6E). These genetic and biochemical validations confirm that NNAT serves as a direct post-transcriptional target of miR-339a, positioning it as a foundational molecular effector within the wider circNID2 regulatory network.

## 4. Discussion

The development of adipose tissue constitutes a critical biological foundation determining the production performance and meat quality of beef cattle, with intramuscular fat deposition directly influencing the marbling, tenderness, and flavor profile of beef. Adipose tissue development is a sophisticated, multi-phasic orchestration requiring the seamless integration of preadipocyte proliferation, lineage-specific differentiation, and programmed cell death. This complex physiological progression is rigorously fine-tuned by a hierarchical regulatory framework encompassing master transcription factors, nuanced signaling cascades, and the multifaceted post-transcriptional control exerted by non-coding RNAs (ncRNAs) [16,17]. Recently, circRNAs have come to the forefront as vital regulators governing lipid deposition. In the bovine model, these non-coding molecules exhibit prominent time- and tissue-specific expression patterns during adipogenesis [18]. Here, we characterized circNID2 as a novel circular transcript derived from the NID2 locus that appears to primarily function as a non-coding RNA involved in bovine adipogenesis. Mechanistically, our data support a potential role for circNID2 as a competing endogenous RNA (ceRNA) that can sequester miR-339a, thereby regulating the inhibitory effect of miR-339a on its target gene NNAT to participate in preadipocyte fate determination. By defining this potential circular regulatory circuitry, these insights advance our conceptual framework of livestock multi-omics, providing a valuable theoretical reference to decrypt the complex molecular networks potentially influencing fat deposition and structural lipid architecture in beef cattle.

The structural characteristics of circular RNAs, including enhanced stability compared with linear transcripts, may contribute to their persistence within cells. In this study, we verified that circNID2 is generated via the back-splicing of exons 3 and 9 of the NID2 gene, with its circular configuration robustly validated through Sanger sequencing and RNase R resistance assays. circNID2 displayed greater stability than its linear counterpart, indicating resistance to exonucleolytic degradation [19]. Furthermore, our expression profiling revealed that circNID2 levels increase continuously throughout the course of bovine adipocyte differentiation. This dynamic expression pattern strongly mirrors previously characterized pro-adipogenic circRNAs, such as circEZH2 and circUBE2Q2, both of which have been documented as pivotal drivers of bovine adipogenesis [20,21]. The upregulation of circNID2 during adipocyte differentiation suggests that circNID2 may be associated with this process. Additionally, this progressive accumulation might be post-transcriptionally enhanced by m^6^A modifications, which could prolong the half-life and structural stability of circNID2 during adipogenesis. However, further functional experiments are required to determine whether it directly regulates adipocyte development.

Our functional experiments revealed that circNID2 affects three key processes: it inhibits proliferation, reduces apoptosis, and promotes differentiation. The transition from proliferation to differentiation usually requires cells to exit the cell cycle. Therefore, inhibiting excessive proliferation is necessary for terminal differentiation [4]. In our study, circNID2 decreased the expression of CDK2, CDK4, Cyclin D1, and Cyclin E1. It also reduced cell activity, indicating an inhibition of the G1/S phase transition. Meanwhile, circNID2 up-regulated key adipogenic genes like PPARγ, CEBPα, and FABP4, leading to increased lipid accumulation. Similar effects have been observed in other circRNAs like circPPARA and circPPARGs [22,23]. Additionally, circNID2 reduced the expression of Bax and p53, helping to maintain adipocyte survival and homeostasis.

Emerging evidence increasingly challenges the canonical non-coding paradigm of circular transcripts, revealing that select circRNAs harbor translatable open reading frames capable of generating functional micropeptides, thereby expanding the functional boundaries of these structural loops during metabolic remodeling [24,25]. Evidence indicates that circARHGAP10 participates in muscle development through its encoded protein, ARHGAP10-202aa. This specific protein binds to MYL6, a molecular event that successfully promotes myoblast differentiation and triggers faster muscle regeneration in vivo [26]. circZNF609 encodes the circZNF609-232aa protein, which inhibits myoblast proliferation while promoting myogenic differentiation [27]. Concurrently, in the realm of adipose development, porcine circANKRD17 has been found to encode a 571-amino acid protein that acts as an upstream activator of the PPAR signaling pathway [24]. This molecular activation subsequently triggers adipocyte differentiation, enhances fatty acid transport and metabolism, and drives lipid droplet formation, thereby exerting a positive regulatory control over intramuscular fat metabolism. However, most circRNAs still function in a non-coding manner. Although we identified a potential ORF in circNID2, we did not detect any protein product through Western blot or staining. This confirms that circNID2 does not have actual translation capacity. This result is consistent with other fat-related circRNAs, such as circINSR and circSAMD4A, which primarily regulate target genes via ceRNA mechanism [28,29].

The most well-established functional archetype for circular RNAs is their capacity to act as ceRNAs that sponge miRNAs. As the application of ceRNA theory expands within lipid biology, an escalating number of studies have verified that circRNAs dictate fat deposition by derepressing key lipogenic targets from miRNA-mediated silencing. Our findings provide additional evidence supporting a potential circNID2/miR-339a/NNAT regulatory relationship during bovine adipogenesis [30]. Our study confirmed the direct binding between circNID2 and miR-339a. While miR-339a over-expression promotes proliferation and inhibits differentiation, circNID2 can reverse these effects. These findings suggest that circNID2 may function as a molecular sponge for miR-339a in bovine adipocytes. Although miR-339 has been studied in tumor cells, its role in adipocytes was previously unclear [31]. Our study is the first to identify miR-339a as a negative regulator of bovine adipogenesis.

NNAT was originally characterized as a neurodevelopmental factor. Recent studies have uncovered its important functions in adipocyte differentiation, insulin sensitivity, and energy metabolism [32,33,34]. Mechanistically, NNAT enhances endoplasmic reticulum Ca^2+^ signaling and activates the PI3K/AKT pathway. These events ultimately promote the expression of adipogenic genes [34,35]. In the present study, NNAT was identified as a direct target of miR-339a, an interaction formally confirmed by dual-luciferase reporter assays demonstrating that miR-339a binds to the 3′UTR of NNAT. Functional experiments further demonstrated that miR-339a significantly represses NNAT expression. We previously showed that circNID2 functions as a molecular sponge for miR-339a. Based on these findings, we propose a potential regulatory model involving circNID2, miR-339a, and NNAT. circNID2 sequesters miR-339a, thereby relieving the inhibition of NNAT. This derepression leads to elevated NNAT expression and ultimately facilitates adipocyte differentiation.

**Figure 7:**
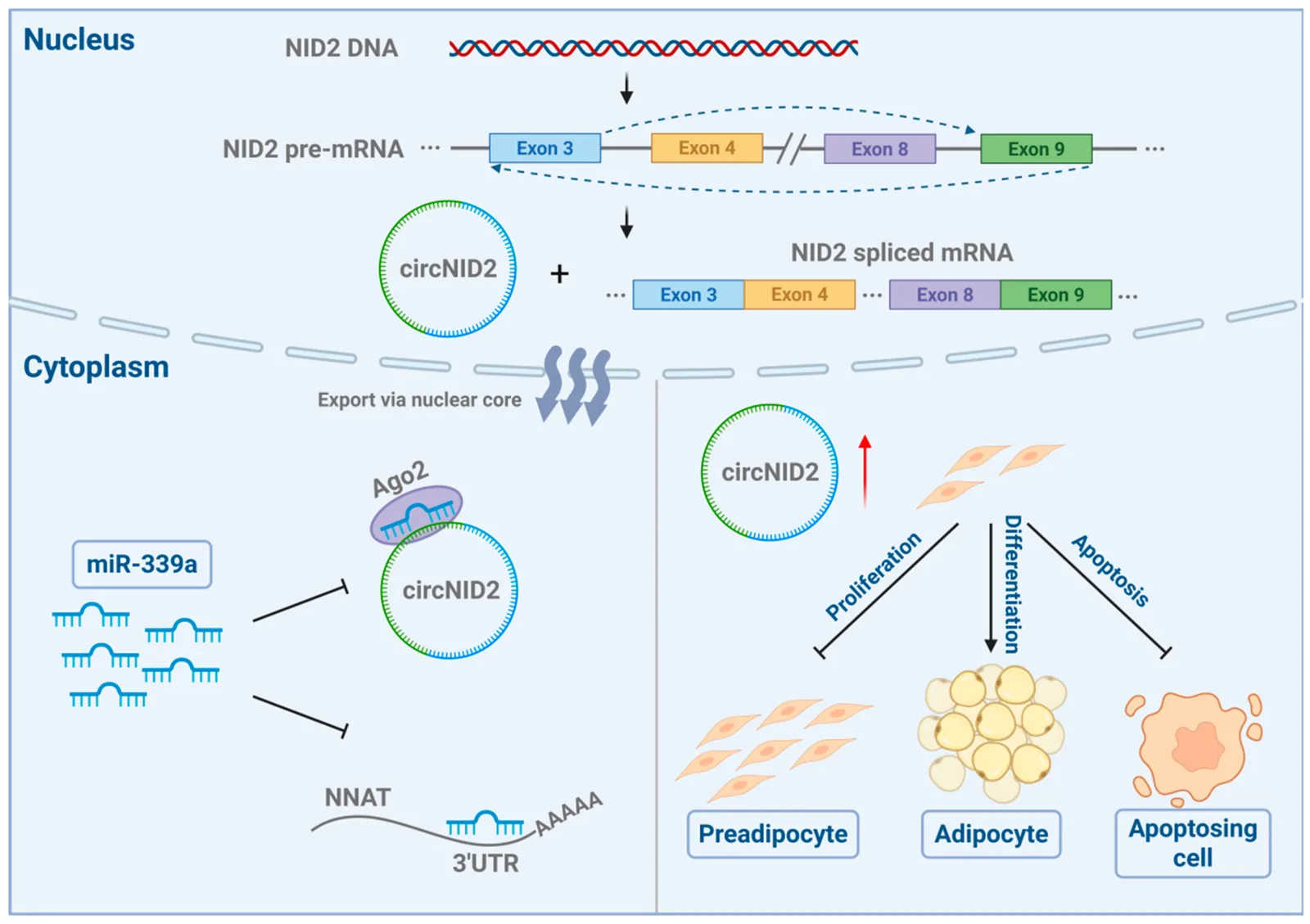
The circNID2/miR-339a/NNAT axis regulates proliferation, differentiation and apoptosis of bovine preadipocytes.

In conclusion, we propose a new regulatory model. During bovine preadipocyte differentiation, circNID2 expression rises and competitively binds to miR-339a. This reduces the inhibitory effect of miR-339a on NNAT, leading to higher NNAT expression. The upregulation of NNAT then activates adipogenic programs, promotes lipid deposition, and ensures adipocyte maturation (Figure 7). Collectively, our results define a circNID2/miR-339a/NNAT cascade that governs adipogenesis via post-transcriptional regulation.

## 5. Conclusions

In summary, this study identified and biochemically characterized circNID2, a stable, ubiquitous circular RNA generated via the non-canonical back-splicing of exons 3 and 9 of the bovine NID2 locus. Despite harboring multiple putative open reading frames, empirical evidence confirmed that circNID2 primarily functions as a non-coding RNA based on the current experimental evidence whose accumulation is synchronized with the chronological stages of preadipocyte commitment. Phenotypic mapping demonstrated that circNID2 acts as a dual-functional homeostatic switch, systematically anchoring preadipocyte population dynamics by suppressing both active proliferation and programmed decay, while driving accelerated lineage differentiation and neutral lipid deposition.

Mechanistically, our findings substantiate a competitive endogenous RNA (ceRNA) model wherein circNID2 acts as an upstream molecular sink. By physically trapping miR-339a, it effectively dismantles the post-transcriptional silencing network normally imposed on NNAT, an interaction fully corroborated by functional rescue metrics. This work establishes a mechanistic framework for circNID2 in fat development, providing a basis for future functional inquiries.

## Figures and Tables

**Table 1:** Primer sequences.

Genes	Primer Sequences (5′→3′)	Product Size/bp
Convergent Primer	F: TTTCCAGGCAGTGTTGGCAT	464
	R: ATTCTTTGGGGCGTGTTCCA	
circNID2	F: GTGACCTGCACAAGTACCAGA	180
	R: CTGGAGCTGGACGTTGTAAGA	
NID2	F: TTCCTTATGGCAGCACAGGAG	202
	R: TAATATCCAGGTTGGCAGCGG	
β-actin	F: CATCCTGACCCTCAAGTA	91
	R: CTCGTTGTAGAAGGTGTG	
CDK2	F: CCCTGGATGAAGATGGACGG	126
	R: GGTGAGGTACTGGCTTGGTC	
CDK4	F: GTGACAAGTGGTGGGACAGT	168
	R: GATACAGCCAACGCTCCACA	
CCND1	F: CCGTCCATGCGGAAGATC	108
	R: CAGGAAGCGGTCCAGGTAG	
PCNA	F: TCCAGAACAAGAGTATAGC	94
	R: TACAACAGCATCTCCAAT	
PPARγ	F: AGGATGGGGTCCTCATATCC	121
	R: GCGTTGAACTTCACAGCAAA	
C/EBPα	F: TGGACAAGAACAGCAACGAG	130
	R: TTGTCACTGGTCAGCTCCAG	
FABP4	F: GTGTCACTGCCACCAGAGTT	76
	R: GGAGTTCGATGCAAACGTCA	
Bcl2	F: ATGTGTGTGGAGAGCGTCAA	143
	R: GGGCCATACAGCTCCACAAA	
Bax	F: GAGATGAATTGGACAGTAACA	118
	R: TTGAAGTTGCCGTCAGAA	
BAD	F: GTCTCCTTCAAGGGGCTTCC	121
	R: AGCCTCCTCTCCCCAAGTTC	
caspase9	F: TGGTGGTCATCCTGTCTC	76
	R: CATCCATCTGTGCCATAAAC	
NNAT	F:AGATCTGGACCAAGTCGGGA	143
	R: CCATTCTGCTGGCTGGTGTA	
TMEM79	F: ATACTGGCTGACCTTCGCTG	157
	R: GGCTCCACCACGAACATGTA	
COPZ1	F: TGACTTGTCATGCAGTCCCC	98
	R: GAGAAGGAACGGGAAGTGCA	
NFIC	F: TCGCCTACACCTGGTTCAAC	220
	R: TTGACTTCGGCCTTCTCACC	
GAPDH	F: GGTCACCAGGGCTGCTTTTA	144
	R: CCGTTCTCTGCCTTGACTGT	
miR-339a-RT	GTCGTATCCAGTGCAGGGTCCGAGGTATTCGCACTGGATACGACGTGAGC	
miR-339a	CGTCCCTGTCCTCCAGGA	
miR-2428-RT	GTCGTATCCAGTGCAGGGTCCGAGGTATTCGCACTGGATACGACACCTCC	
miR-2428	CGTGGCTGGAGTGCGGC	
miR-180c-RT	GTCGTATCCAGTGCAGGGTCCGAGGTATTCGCACTGGATACGACAAACTC	
miR-180c	CGAACATTCAACCTGTCGGT	
miR-12039-RT	GTCGTATCCAGTGCAGGGTCCGAGGTATTCGCACTGGATACGACAGAGAA	
miR-12039	CCAGGGCCCTGGCTTC	
miR-2461-3p-RT	GTCGTATCCAGTGCAGGGTCCGAGGTATTCGCACTGGATACGACAGGCAC	
miR-2461-3p	CGCGTCAGACTGAGAGCAGT	
miR-R	AGTGCAGGGTCCGAGGTATT	
U6	F: CGAAACCACCACCACACT	
	R: GGATATGGACTGACTCAGGA	

## Data Availability

The raw data supporting the conclusions of this article will be made available by the authors on request.

## Supplementary Materials

The following supporting information can be downloaded at: https://www.mdpi.com/article/10.3390/biom16081113/s1. The original Western blot images are available in the Supplementary Materials.

## Abbreviations

The following abbreviations are used in this manuscript:

IMF	intramuscular fat
ncRNAs	non-coding RNAs
circRNAs	circular RNAs
miRNA	microRNA
ceRNA	competing endogenous RNA
NID2	nidogen 2
NNAT	Neuronatin
ORF	open reading frames
RT-qPCR	Real-time Quantitative polymerase chain reaction
EdU	5-ethynyl-2′-deoxyuridine
