# Supplementary material for: Circular RNA circNID2 Promotes Bovine Adipogenesis via miR-339a/NNAT
Source: Biomolecules. 2026 Jul 30;16(8):1113. doi: 10.3390/biom16081113 (PMC13511229; doi:10.3390/biom16081113)
Supplement: Supplementary file 1 [file biomolecules-16-01113-s001.zip › biomolecules-4410405-supplementary.pdf]

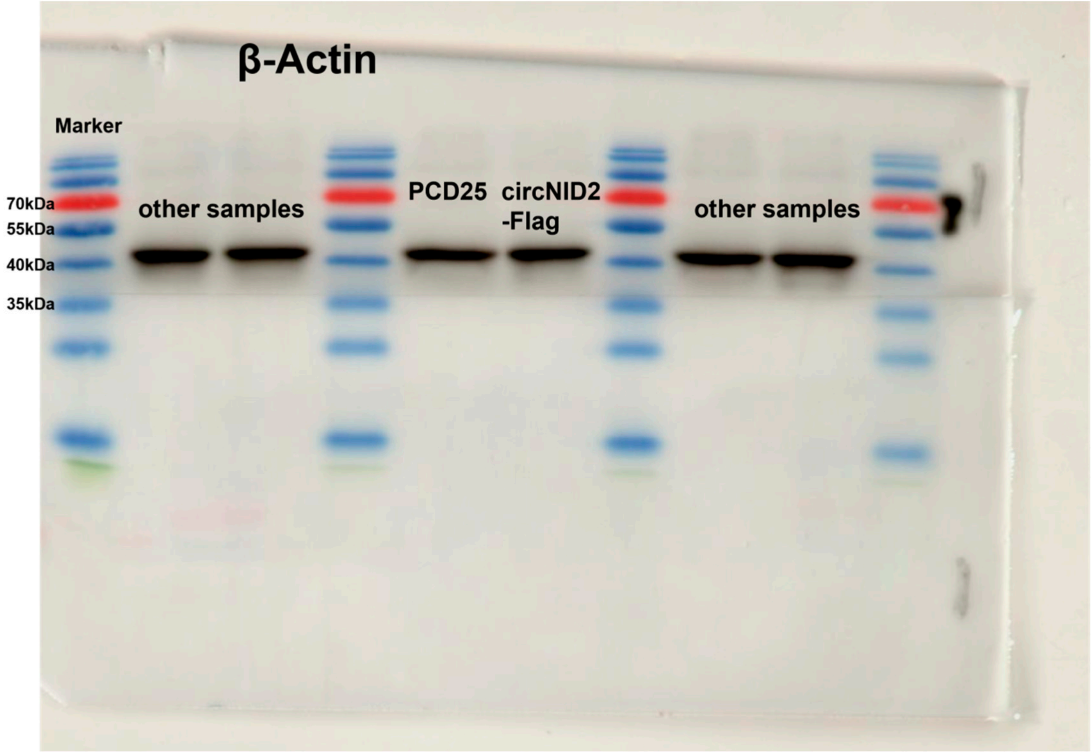
β-Actin
Marker
70kDa
55kDa
40kDa
35kDa
other samples
PCD25
circNID2
-Flag
other samples

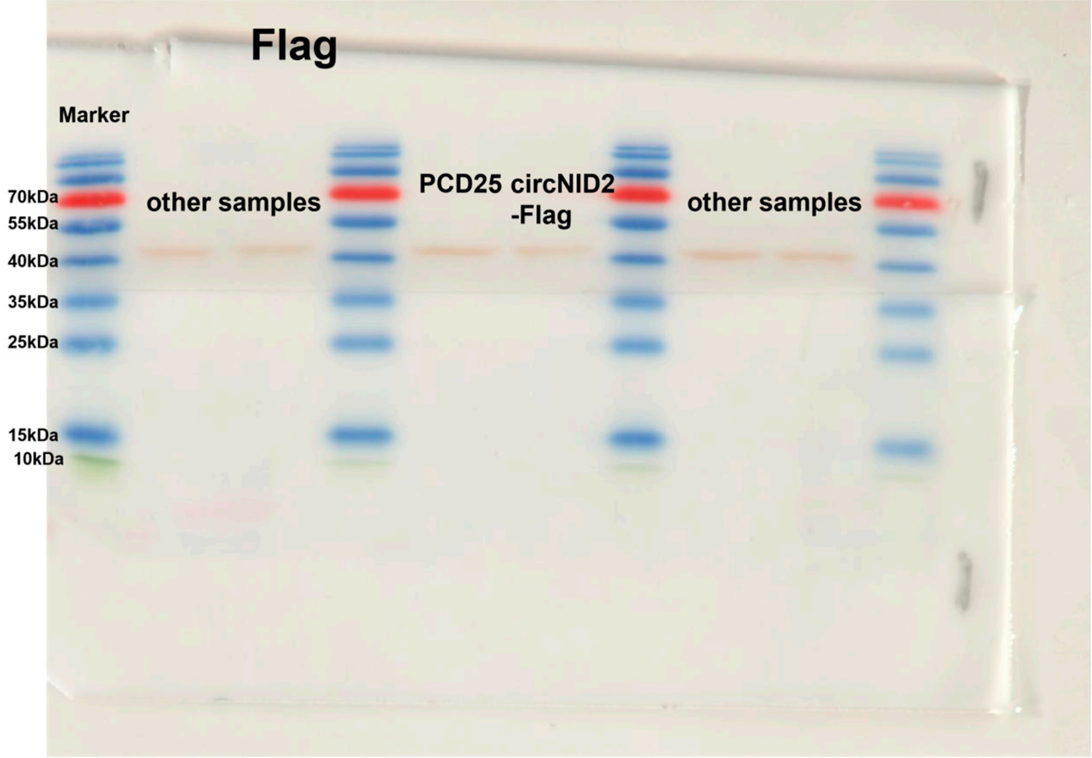
Flag
Marker
70kDa
55kDa
40kDa
35kDa
25kDa
15kDa
10kDa
other samples
PCD25
circNID2
-Flag
other samples
